# Effect of ultrasound combined with chemical pretreatment as an innovative non-thermal technology on the drying process, quality properties and texture of cherry subjected to radio frequency vacuum drying

**DOI:** 10.1016/j.ultsonch.2024.106980

**Published:** 2024-07-04

**Authors:** Zepeng Zang, Fangxin Wan, Guojun Ma, Yanrui Xu, Bowen Wu, Xiaopeng Huang

**Affiliations:** College of Mechanical and Electrical Engineering, Gansu Agricultural University, Lanzhou 730070, China

**Keywords:** Cherry, Radio frequency vacuum drying, Ultrasound, Chemical pretreatment, Bioactive compounds

## Abstract

To obtain high-quality cherry products, ultrasound (US) combined with five chemical pretreatment techniques were used on cherry prior to radio frequency vacuum drying (RFV), including carboxymethyl cellulose coating (CMC), cellulase (CE), ethanol (EA), isomaltooligosaccharide (IMO), and potassium carbonate + ethyl oleate (PC + AEEO). The effect of different pretreatments (US-CMC, US-CE, US-EA, US-IMO, US-(PC + AEEO)) on the drying characteristics, quality properties, texture, and sensory evaluation of cherries was evaluated. Results showed that the dehydration time and energy consumption were decreased by 4.17 − 20.83 % and 3.22 − 19.34 %, respectively, and the contents of individual sugars, soluble solid, total phenolics (TPC), natural active substances, total flavonoids (TFC), and antioxidant properties (DPPH, ABTS and FRAP) were significantly increased after US combined with five chemical treatments (*P* < 0.05). Moreover, the pretreatment played important role in improving texture properties and surface color retention in the dried cherries. According to the sensory evaluation analysis, the dehydrated cherries pretreated with US-CMC exhibited the highest overall acceptance, texture, crispness, color, and sweet taste showed lower off-odor, bitter taste and sour taste compared to control and other pretreatments. The findings indicate that US-CMC pretreatment is a promising technique for increasing physicochemical qualities and dehydration rate of samples, which provides a novel strategy to processing of dried cherry.

## Highlights

• Combination of pretreatments technologies improved drying rate by 10.34–41.38%.
• US-CMC pretreatment was the best combination on improving quality properties.
• US-CMC + RFV drying exhibited the highest overall acceptance and better texture.
• Individual sugar and organic acids exhibited antagonistic effects during dehydration.
• US combined with chemical pretreatment can reduce energy consumption by 3.22−19.34 %.

## 1 Introduction

Cherries (Rosaceae family), mainly native to the temperate and subtropical regions (e.g., Australia, Canada, Chile, China, and Europe), are known as a popular fruit that has gained increasing importance in fruits and vegetables industry due to its high nutritional value, attractive colors and strong aroma [1]. Recently, studies have shown that cherries are rich in bioactive compounds, encompassing amino acids, sugars, total phenolics, total flavonoids, anthocyanins, vitamin C, dietary fibers, organic acids and soluble solids [2], [3]. Those physiochemical compounds possess the function of antitumor, anti-aging, prevent chronic diseases and cardioprotective, making them suitable for developing value-added products [4]. Freshly harvested cherry are prone to rot and difficult to preserve due to their high moisture contentand susceptible to moisture loss and microbial reproduction in the transportation, processing and storage process [5]. While fruit and vegetable cells also have a high rate of biochemical reactions, making the deterioration of the quality of cherries accelerated, resulting in economic losses and environmental problems [6]. Drying is an energy-intensive preservation technique that not only effectively inhibits water activity, enzymatic degradation, and microbial growth, prolonging the shelf life, but also imparts unique texture and flavor to the samples, increasing the comprehensive utilization rate and added value of fruits and vegetables [7]. Nevertheless, the traditional heating method mainly transfers heat from the external heat source to the internal material through heat conduction and heat convection, which results in longer heating time and poorer heating uniformity, thus causing the loss of flavor, color and nutrients of fruits and vegetables [8]. Therefore, it is necessary to develop the new high-efficiency combination drying technology to fulfill the requirements of energy-saving and high-efficiency, green safety, low-carbon and high-quality products.

Radio frequency (RF) is a high-frequency electromagnetic waves with a frequency range of 10–300 MHz. Its mechanism relies on the action of water molecules under high-frequency electric fields, inducing friction among polar molecules, leading to volumetric heating and subsequent water removal from the material [9], [10]. RF combined with vacuum drying prevents thermal and oxidative damage to natural active compounds at sub-atmospheric pressure and reduces water's boiling point. Compared to the traditional dehydration approach, radio frequency vacuum (RFV) technology offers advantages such as preserving heat-sensitive compounds, low energy consumption, and uniform heating [11], [12]. As a sustainable drying technology, RFV drying technology is widely applied in agriculture products and food industry, such as kiwifruit [13], apple slices [14], and inshell hazelnuts [15]. Currently, RFV technology is mainly applied to disinfestation and pasteurisation/sterilisation of cherries. Monzona et al. [16] investigated the effect of RFV-heating treatments on the quality of “Bing” cherries fruits and the mortality of codling moth larvae, and found that higher quality was obtained from RFV treatment. Tang et al. [17] evaluated the effect of RF-hot water treatments on the sweet cherries' codling moth control effects. Nonetheless, the influence of RFV technology on the drying and physiochemical properties of cherry have not yet been reported. Additionally, studies have shown that the RFV drying approach has some drawbacks, such as thermal deviation during heating and edge effects, as well as the occurrence of enzyme-catalyzed browning, which significantly decrease the nutritional value of the dried products [18]. To address these challenges and strengthen the dehydration process, researchers have explored various pretreatment methods for dried fruits and vegetables such as cold plasma pretreatment [19], high-humidity hot air impingement blanching [20], vacuum-steam pulsed blanching [21], ozone pretreatment [22], and ultrasound (US) pretreatment [23].

As a potential non-thermal technique, US pretreatment is gaining enormous attention in the various fruits and vegetables processing industry [24], [25]. Ultrasonic interact with the medium to generate thermal, mechanical, and cavitation effects, thereby enhancing the drying process of materials and improving the effective moisture diffusivity. Among them, the thermal effects cause the continuously absorption of ultrasonic energy by the material, so that the temperature of the material rises. Mechanical effects cause the material to be repeatedly compressed and stretched, resulting in the structural forces surpassing the surface adhesion forces of moisture, thereby facilitating the removal of moisture from the material [26], [27], [28]. The cavitation effect is the result of cavitation bubbles forming, growing, and collapsing under the action of ultrasonic waves. This process eventually generates microjet and instantaneous high temperature and high pressure in the local area, breaking the solid–liquid heat and mass transfer resistance, and improving the dehydration efficiency [29]. Research have shown that US treatment can enhance the flavour and consumer acceptability of agricultural products, possibly due to the cavitation effect of US, leading to the release of phenolics, aldehydes and esters compounds [30]. It has been observed that US could dramatically improve the retention of some bioactive phytochemicals in *Angelica sinensis* [31]. Similar result was found by Wang et al. [32] on “*Actinidia deliciosa*” kiwifruit slices. Additionally, US has a positive impact in reducing the energy consumption and improving the efficiency of heat and mass transfer in the dehydration process. For example, it has been observed that US pretreatment can improve the drying kinetics and heat and mass transfer of carrot discs [33], banana slices [26], and potato slices [34].

The surface of cherries is covered with a dense waxy layer that is highly hydrophobic, which severely hinders the migration of water within the sample. Therefore, various chemical pretreatments can be applied to dissolve and destroy the waxy layer, thereby increasing the drying rate [35]. In recent years, carboxymethyl cellulose (CMC), as a kind of edible coating with the advantages of biodegradable, safety and non-toxicity, lower cost, convenient operation, and better preservation effect, has attracted the attention of researchers [36], [37]. It creates an improved gas environment around the fruit, minimizes the loss of heat-sensitive compounds and delays the respiration rate of the fruit to achieve the effect of extending the shelf life [38]. Furthermore, ethanol (EA) is an organic compound with a lower boiling point and surface tension than water [39]. A mixture of carbonate + ethyl oleate (PC + AEEO) can alter the ultrastructure of the waxy layer. Isomaltooligosaccharide (IMO) and cellulase (CE) has a significant advantage in increasing cell membrane permeability [40], [41]. However, few researches have focused on the application of US combined with the above chemical pretreatment techniques in fruits and vegetables processing.

To date, previous researches have primarily focused on the impact of individual drying and conventional pretreatments during dehydration on the rehydration properties and microstructure of cherries. Nevertheless, the research on the impact of US, US combined with chemical pretreatment, and especially US combined with edible coatings on the quality of cherry during RFV drying is limited. Here, the feasibility of US-CMC, US-CE, US-EA, US-IMO and US-(PC + AEEO) as pretreatment approaches to enhance the drying characteristics, quality properties, and overall acceptance of RFV dried cherries were investigated.

## 2 Materials and methods

### 2.1 Experimental materials

Fresh cherries (*Prunus pseudocerasus* (Lindl.) G. Don) with an initial moisture level of 89.62 ± 1.0 % (w/w, wet basis) were purchased from Tianshui city, and promptly placed in cold storage at 2-4℃. To guarantee the dependability of the results, cherries with consistent size (10–15 g each sample) and undamaged shape were selected as experimental materials. The 200 g cherries were vertically cut into half using a fruit and vegetable slicer and kept to stabilize at room temperature (25℃) for 1 h before each test. To prevent oxidative browning of cherries, the cherries were soaked in 1.50 % citric acid solution for 5 min to achieve the purpose of color protection.

### 2.2 Pretreatment procedure

**Fig. 1:**
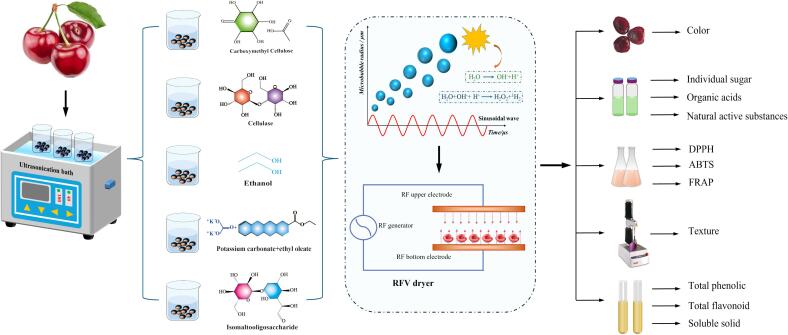
Illustrative diagram of US-CMC + RFV, US-CE + RFV, US-EA + RFV, US-IMO + RFV, and US-(PC + AEEO) + RFV treatment of cherry.

The specific treatments steps and quality-determination methods for US-CMC, US-CE, US-EA, US-IMO and US-(PC + AEEO) are shown in Fig. 1:

US + CMC pretreatment: Carboxymethyl cellulose was dissolved in deionized water at 70 °C, and CMC was slowly added to the water with constant stirring to obtain a transparent solution of CMC film with a mass fraction of 1.5 % (g/mL). The cherries were uniformly submerged in the coating solution, and then placed them into an US for 20 min (25℃, 180 W power, 40 kHz frequency).

US + EA pretreatment: The 200 g cherries were submerged in ethanol (99.8 % v/v), and then also placed them into an US bath for compound pretreatment.

US + IMO pretreatment: The concentration of 30 % isomaltooligosaccharide solution was prepared as the osmotic solution. The permeation temperature was 25℃, the material-liquid ratio was 1:5 g/L, and the pretreatment should be stirred sufficiently to overcome the mass transfer resistance generated by IMO solution in the osmosis process, and then the beaker with submerged cherries was placed into an US bath for compound pretreatment.

US+(PC + AEEO) pretreatment: The PC + AEEO solution was prepared at 25℃ with slightly modified parameters according to the method of An et al [42]. PC + AEEO was prepared from 2 % potassium carbonate and 1 % ethyl oleate. Then US composite pretreatment was performed.

US + CE pretreatment: Cherries were placed in a cellulase enzymatic solution with a mass fraction of 1 % and the beaker containing the enzymatic solution and cherries was placed in an US bath for compound pretreatment.

### 2.3 Radio frequency vacuum drying

RFV drying (Control): after US-CMC, US-CE, US-EA, US-IMO, and US-(PC + AEEO) pretreatments, the cherries were uniformly spread in a single layer on a polystyrene container, and then the samples were dried in a RFV equipment. The plate spacing of 90 mm, the vacuum degree of 0.035 MPa, and the drying temperature of 55℃ were selected as the RFV drying conditions. Measurements were taken every 30 min until the samples reached a final moisture content of 15 % wet basis.

### 2.4 Moisture content and drying rate

The moisture ratio (*MR*) and drying rate (*DR*) of the cherries were computed using Eq. (1), (2) [43]:

(1) $$\mathrm{MR}=\left(M_{t}-M_{e}\right)/\left(M_{o}-M_{e}\right)$$

(2) $$\mathrm{DR}=\left(M_{t}-M_{t-\Delta t}\right)/\Delta t$$

where *M*_t_ is the dry base moisture content at the drying time t, g/g; *M*_e_ is the equilibrium water content, g/g; *M*_0_ is the initial dry basis moisture content, g/g.

### 2.5 Energy consumption

Two separate watt-hour meters (ADL200/C, Acrel Electric Co., Ltd. Shanghai, Chian) were used to individually measure the energy consumption of the US pretreatment and RFV drying processes. The total energy consumption was computed with Eq. (3):

(3) $$\mathrm{TEC}=E_{\left(US\right)}+E_{\left(RFV\right)}$$

where *E*_(US)_ and *E*_(RFV)_ is the energy consumption of US pretreatment and RFV dehydration processes, respectively.

### 2.6 Shrinkage ratio

The shrinkage ratio of the cherries were computed using Eq. (4):

(4) $$\mathrm{SR}=\left(V_{0}-V\right)/V_{0}\times 100\%$$

where *V*_0_ and *V* represent the volume of cherries before and after drying (mL), respectively.

### 2.7 Color measurement

The apparent colors of the cherries were determined by colorimeter, and the L* (lightness, 0 − 100), a* (redness, −60 − 60), and b* (yellowness, −60 − 60) were recorded [44], [45]. The centre of the cherry was used as test area, and the total colour difference (ΔE) and the browning index (BI) were computed using Eq. (5), (6):

(5) $$\Delta E=\sqrt{{\left(L^{*}-L_{0}\right)}^{2}+{\left(a^{*}-a_{0}\right)}^{2}+{\left(b^{*}-b_{0}\right)}^{2}}$$

(6) $$\mathrm{BI}=\left(x-0.3/0.17\right)\times 100$$

where L*, a*, b* represent the brightness/darkness, redness/greenness, yellowness/blueness of dried cherries, whereas L_0_, *a*_0_, *b*_0_ represent the same for fresh cherries.

### 2.8 Determination of individual sugar, organic acids and natural active substances content

#### 2.8.1 Preparation of extract

The cherry powder was extracted with 75 % methanol (v/v; 25 mL) using US (200 W, 60 kHz, 25℃) for 30 min. The solution was centrifuged (4℃, 12,000 r/min) for 15 min. The supernatant was then collected to measure the physicochemical qualities of the cherries.

#### 2.8.2 Analysis of organic acids, individual sugars content and natural active substances

The organic acids, individual sugars, and natural active substances were determined at 250 nm, 280 nm, and 248 nm using a high performance liquid chromatography system (Agilent 1100, Agilent Technology Co., Ltd, America). The chromatographic column was XDB-C_18_ (250 mm 4.6 mm, 5 μm) [46]. Briefly, the experimental conditions were:

Organic acids: the gradient elution was performed at a flow rate of 1.0 mL/min using 0.1 % phosphoric acid solution (A) and acetonitrile (B): 0 − 2 min, 85 % B; 2 − 5 min, 85 − 60 % B; 5–10 min, 60 − 45 % B; 10 − 15 min, 45 − 20 % B; 15 − 18 min, 20 − 75 % B; 18 − 20 min, 75 − 85 % B.

Individual sugars: the gradient elution was performed using acetonitrile (A) and water (B): 15 %−22 % B, 0 − 8 min; 22 − 32 % B, 8 − 12 min; 32 − 48 % B, 12 − 20 min; 48 %−65 % B, 20 − 24 min; 65 − 15 % B, 24–26 min.

Natural active substances: the analytical method outlined by Hayaloglu et al. [47] was adopted for the analysis of natural active substances. Specifically, the gradient elution was performed at a flow rate of 1 mL/min using 0.2 % formic acid (B) and acetonitrile (A): 0 − 7 min, 90 − 85 % B; 7 − 10 min, 85 − 84 % B; 10 − 16 min, 84 − 80 % B; 16 − 22 min, 80 − 78 % B; 22 − 25 min, 78 − 70 % B; 25 − 30 min, 70 − 95 % B; 30 − 32 min, 95–95 % B.

### 2.9 Determination of total soluble solid content

After rehydrating the dried cherries, the total soluble solid contents of the cherries were determined using a PAL-1 handheld refractometer (ATAGO Scientific Instrument Co., Ltd, Tokyo, Japan).

### 2.10 Determination of texture

Textural properties were determined using a Ta.XT 2i/50 texture analyzer [48]. The P/36R probe was used, and test conditions were set as follows: the pre-test speed was 2.0 mm∙s^−1^, the test speed was 1.0 mm∙s^−1^, the post-test speed was 5.0 mm∙s^−1^, and the compression ratio was 60 %. Each indicator was measured 12 times and the average value was taken after removing the maximum and minimum values.

### 2.11 Determination of TPC and TFC

The TPC and TFC were quantified using the Folin-Ciocalteu reagent method and NaNO_2_-AlCl_3_-NaOH method, respectively, following the protocol outlined by Jiang et al [49] with minor adjustments. The specific experiment steps are as follows:

Total phenolic determination: 200 μL of the sample extract was placed in a test tube. Immediately, 2.0 mL of 10 % Folin-Ciocalteu reagent and 1.0 mL of 7.5 % Na_2_CO_3_ solution were added and completely mixed. The absorbance was measured at 760 nm by a UV–visible spectrophotometer, using a solution without the sample as a blank control.

Total flavonoid determination: 5 μL of the sample extract was placed in a test tube, and 2.0 mL of 90 % phenol solution was added and completely mixed. Then, 2.0 mL of distilled water, 0.3 mL of 5 % NaNO_2_ solution, and 0.3 mL of 10 % AlCl_3_ solution were added and mixed well. Subsequently, the absorbance of the mixed extract was measured at 510 nm, with a blank control containing no sample extract.

### 2.12 Determination of antioxidant activity

The antioxidant activities in cherry extracts were measured using the DPPH free radical scavenging activity, ABTS radical scavenging activity, and FRAP ferric ion reducing antioxidant power, according to the method of Zang et al. [26], [48], Yang et al. [50], and Lopes et al. [51] with slight modifications.

### 2.13 Microstructure observation

The surface and internal microstructure of dried cherries were evaluated using a scanning electron microscope (S3400N, Hitachi Corporation, Tokyo, Japan) at 450 × and 500 × magnification, respectively, according to the method of Zang et al [31].

### 2.14 Sensory evaluation

Hedonic sensory analysis was applied to evaluate the flavor attributes and overall preference of all dried samples. Thirty individuals (15 males and 15 females) from GAU who have completed training in sensory evaluation were invited. The age of participants were between 20 and 50 years old. The cherry was assessed for sour taste, sweet taste, bitter taste, color. And other sensory attributes using a 10-point hedonic scale [48]. Each participant's eyes were covered during the measurement and the cherries were randomly placed in trays.

### 2.15 Statistical analysis

Origin 8.5 software and Microsoft Excel 2010 was used to plot and analyze the quality and drying data. The experimental data were analyzed by SPSS Statistics 22.0 software, and the differences between samples were analyzed by one-way analysis of variance (ANOVA).

## 3 Results and discussion

### 3.1 Drying characteristics

**Fig. 2:**
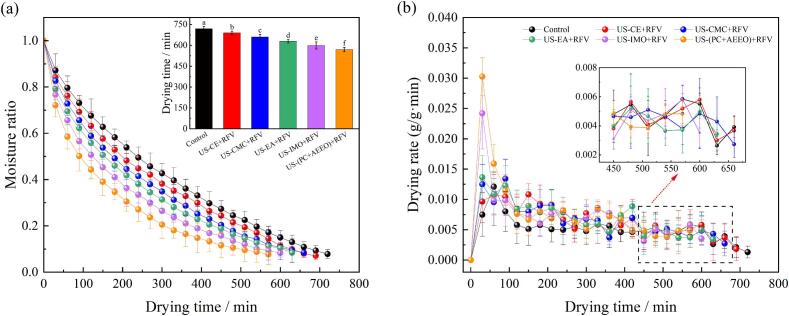
Moisture ratio (a) and drying rate (b) of cherries subjected to various treatment conditions.

The moisture ratio and drying rate of cherries during the drying process under various pretreatment methods are illustrated in Fig. 2. The RFV dehydration time after US-CMC, US-CE, US-EA, US-IMO, and US-(PC + AEEO) pretreatments were 660 min, 690 min, 630 min, 600 min, and 570 min, respectively. Compared with the control (720 min), the dehydration time was reduced by 8.33 %, 4.17 %, 12.50 %, 16.67 %, and 20.83 %, respectively. Additionally, the average dehydration rates improved by 15.52 %, 10.34 %, 20.69 %, 27.59 %, and 41.38 %. Reason may be that the mechanical effect of US generated strong stretching and contraction, which disrupts the internal tissue structure of the cherry, weakened the intermolecular cohesion, and enhanced the turbulence of the internal moisture [52]. Meanwhile, the shock wave, micro-jet and micro-disturbance generated by the cavitation effect of US caused deformation inside the cherry and subsequently lead to interparticle collisions. And it promoted the generation of microfine channels and solid surface activation, reduced the inherent diffusion boundary layer, broke the bond between water molecules and molecules on the solid surface, and decreased the adhesion force of water on the inner wall of the cell, thus accelerating the diffusion process of water, which was beneficial to the evaporation and escape of water in the microfine tubes [53].

**Fig. 3:**
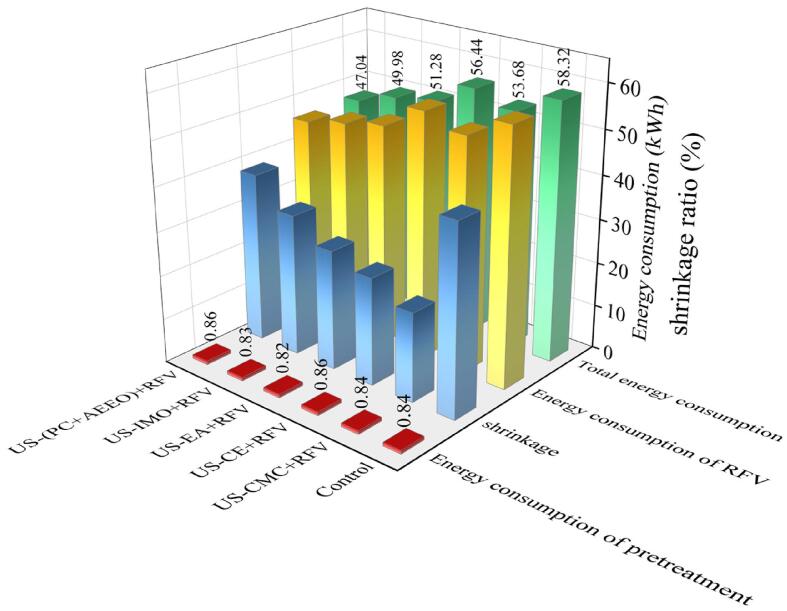
Energy consumption and shrinkage parameters during drying of cherries after ultrasound combined with various chemical pretreatment.

The dehydration time of RFV was reduced 60 min by combining RFV and US-CMC coating pretreatment. However, the result indicated that the CMC coating had no significant effect on the improvement of the drying rate, which was closely related to the lower water vapor permeability and high hydrophilic of the CMC edible coating. This suggests that the CMC coating may have somewhat reduced the absorption of ultrasonic energy by the cherries and hindered the migration of water during the dehydration. The properties limited the efficiency of transferring moisture from the inside to the surface of material during drying [54]. US-CMC pretreatment can shorten RFV dewatering time probably because the mechanical, cavitation and thermal effects of US improve the mass transfer rate of cherries in the dewatering process. Meanwhile, CMC coating had an advantage of forming a hydrocolloid film on the surface of fruits and thereby limiting drying shrinkage and accelerating the drying process (Fig. 3). The results showed that US-(PC + AEEO) and US-EA pretreatment also significantly improved the dehydration efficiency, probably because the PC + AEEO and EA solutions into the cherry cells could promote the dissolution of the surface waxy layer and disrupt the integrity of the epidermal cells and enhance the cell wall and cell membrane permeability (Fig. 6 (A)). Secondly, US treatment can increase different types of cavities and microchannels in cherry cells, which improved their permeability and diffusivity, making it easier for water to diffuse from the pulp cells. Results showed that the pericarp permeability of cherry was also effectively improved due to CE treatment and shortened the drying time.

### 3.2 Energy consumption

Energy consumption is not only an important factor for evaluating the advantages and disadvantages of fruit and vegetable drying process, but also an essential index for measuring the economy, environmental friendliness and technological advancement of the drying process. Efficient drying equipment and process can significantly reduce energy consumption and improve production efficiency. The energy consumption of cherry RFV drying process after different pretreatment is shown in Fig. 3. Energy consumption was reduced by 7.96 %, 3.22 %, 12.07 %, 14.30 %, and 19.34 % after US-CE, US-CE, US-EA, US-IMO, and US-(PC + AEEO) pre-treatments, respectively, compared to the control. It is shown that US combined with chemical pretreatment can reduce energy consumption (3.22 ∼ 19.34 %) and improve drying efficiency (10.34 ∼ 41.38 %) during cherry dehydration. The results showed that US-(PC + AEEO) + RFV exhibited the lowest energy consumption (47.04 kWh), which was attributed to the shortened drying time and increased moisture diffusion rate of the US-(PC + AEEO) pretreatment. Compared with US-CE + RFV, the drying energy consumption of US-CMC + RFV-treated cherries was reduced by 4.91 %, which indicates that US-CMC + RFV can also achieve the goals of energy saving, emission reduction, and cost reduction. Overall, US combined with chemical pretreatment could be considered as an effective approach to improve the quality of the dried cherry fruit and reduce energy consumption.

### 3.3 Color

Color is the first attribute perceived by consumers in fruits and vegetables and is one of the most important evaluation indexes of the sensory quality for cherries. The effects of different pretreatments on the color of cherry are shown in Table 1. The L*, a* and b* values for fresh cherries were 29.78 ± 2.48, 11.40 ± 1.02 and 3.21 ± 0.38. The brightness of all cherries was significantly reduced after dehydration treatment, and there were also significant differences in brightness and color difference between different pretreatment groups (*P* < 0.05) compared with fresh cherries. It was possible that the prolonged exposure of material to high-temperatures and high-humidity increased the possibility of contact of intracellular anthocyanins and polyphenols to oxygen and related enzymes, resulting in significant quality degradation on the surface of the cherries. Furthermore, anthocyanins and proanthocyanidins, which are the main characteristic compounds causing color changes in fruits and vegetables, were highly unstable and susceptible to degradation. Similar results were reported by Tenuta et al. [55] and Nemzer et al [56]. The mechanical action of US causes leakage and degradation of hydrophilic compounds such as anthocyanins. A negative correlation has been reported between changes in cherry color and anthocyanin loss during the dehydration process. Noticeably, the color difference of dried cherry products after US-CE, US-CMC, US-EA, US-IMO and US-(PC + AEEO) pretreatments were significantly decreased in color value and increased in brightness compared with the control. It indicated that US combined with chemical pretreatments played a certain role in color protection during the dehydration of cherries, which could reduce the browning phenomenon during processing and improve the apparent quality of the samples. The lowest ΔE and BI were found in cherry after the US-CMC pretreatment (ΔE = 5.59 ± 1.01, BI = 21.77 ± 2.49), followed by the US-CE pretreatment, and the highest was found in the control. The result could be due to that the CMC and CE coating reduced the oxygen-contact induced oxidation of cherry, thereby reducing enzymatic and nonenzymatic browning, oxidative condensation of polyphenolic compounds, degradation of pigment, and Maillard reaction [37]. US-(PC + AEEO) pretreatment sample had lower L* and higher ΔE value than US-IMO and US-EA sample. The US-(PC + AEEO) pretreatment, although had the shortest dehydration time, simultaneously destroyed the waxy layer and surface structure of the cherries, allowing an aggravated oxidative decomposition of ascorbic acid and phenolic compounds, resulting in a lower color of the sample.

**Table 1:** Color and soluble solid content of cherries subjected to various pretreatment conditions.

Drying conditions	Color					Soluble solid (°Bx)
	L*	a*	b*	ΔE	BI	
Fresh	29.78 ± 2.48^a^	11.40 ± 1.02^a^	3.21 ± 0.38^a^	-	-	16.47 ± 0.94^a^
Control	20.49 ± 3.15^d^	2.45 ± 1.53^d^	1.48 ± 0.12^d^	13.02 ± 2.12^a^	43.50 ± 3.17^a^	9.79 ± 0.81^e^
US-CMC + RFV	27.26 ± 4.21^b^	6.42 ± 1.44^b^	2.92 ± 0.40^b^	5.59 ± 1.01^e^	21.77 ± 2.49^e^	13.79 ± 0.68^b^
US-CE + RFV	25.28 ± 2.79^c^	4.88 ± 1.06^c^	3.04 ± 0.13^ab^	7.92 ± 0.68^de^	25.97 ± 3.04^d^	13.14 ± 0.43^bc^
US-EA + RFV	25.6 ± 3.14^c^	4.24 ± 0.89^cd^	2.08 ± 0.21^cd^	8.37 ± 0.79^d^	33.66 ± 1.97^c^	12.05 ± 0.78^c^
US-IMO + RFV	21.9 ± 3.01^cd^	6.44 ± 1.77^b^	2.14 ± 0.27^c^	9.37 ± 1.24^c^	32.24 ± 2.58^c^	11.84 ± 0.59^d^
US-(PC + AEEO) + RFV	20.58 ± 1.59^d^	6.45 ± 1.01^b^	2.09 ± 0.20^cd^	10.51 ± 1.37^b^	36.53 ± 1.69^b^	11.69 ± 0.72^d^

### 3.4 Individual sugars and organic acids

**Fig. 4:**
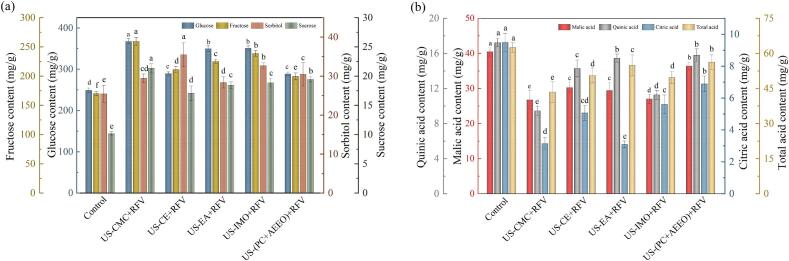
Individual sugars (a) and organic acids content (b) of cherries subjected to various pretreatment conditions.

The concents of individual sugars and organic acids in cherries subjected to various treatments are illustrated in Fig. 4. The interaction of US combined with various chemical pretreatmens had a statistically significant effect on the sugars and acids content of cherry during dehydration. The highest concents of glucose (367.57 ± 6.12 mg/g), fructose (259.39 ± 5.84 mg/g), and sucrose (21.33 ± 1.56 μg/g) were observed in cherries pretreatment with US-CMC, which were 1.48, 1.53, and 2.09 times higher than that of the control, respectively. Specifically, it is likely attributable to the formation of a dry CMC crust on the surface of the cherries, which could increase resistance to individual sugars diffusion and counterbalance the effects of surface etching. The sucrose content of cherries increased by 3.08 % after US-(PC + AEEO) pretreatment compared to US-IMO. This may be due to the higher osmotic pressure in the US-IMO pretreatment induced more sucrose molecules to penetrate into the cherry tissue. Some of these sugar molecules enter the cytoplasm and interact with components in the raw material to form hydrogen bonds or intermolecular forces, while the remaining sugar molecules were adsorbed on the surface of the cell wall to form a hardened layer hindered the exudation of organic acids to a certain extent. Research indicates that glucose and fructose are produced because sucrose invertase catalyzes the irreversible hydrolysis of sucrose. The lower individual sugars contents in cherries after US-(PC + AEEO) pretreatment may be due to the fact that PC + AEEO treatment increased cell permeability and decreased the protection of the sample epidermis against sugars compounds. Malic acid and citric acid constitute the principal acidic components attributed to the sensory perception of cherries, playing a pivotal role in shaping the taste and aroma profiles of the samples, similarly reported by Nowicka et al [57]. The US-CMC samples showed the lowest malic acid and citric acid content, while the highest content of total acid (62.48 ± 3.43 mg/g) was found in control samples. A sharp decrease in quinic acid was observed in the dried samples with US-EA and US-CE pretreatments, due to the application of US combined with EA and CE promoted the release of acidic compounds such as quinic acid by disrupting cell walls and cell membranes.

### 3.5 Natural active substances

Catechin, chlorogenic acid, neochlorogenic acid, isochlorogenic acid, cynaroside, quercetin, kaempferol are the primary bioactive compound of cherry, which have many health benefits including antioxidant, anti-cancer, cardioprotective and treating Alzheimer's disease. The various pretreatments groups exerted a statistically significant influence on the alteration of natural active substance levels in cherries, with chlorogenic acid, neochlorogenic acid, and catechin exhibiting notably higher levels compared to other natural active substances. Based on the evidence from Table 2, the contents of neochlorogenic acid (241.48 ± 4.12 mg/100 g) and isochlorogenic acid (23.79 ± 1.03 mg/100 g) were observed to reach their peaks in the samples treated with US-CE, exhibiting increases of 1.25 and 1.48 times, respectively, relative to the control. This indicated that US-CE coating pretreatment was effective and feasible technique to improve neochlorogenic acid and isochlorogenic acid retention in cherry. US-CMC processed dried cherry products showed similar results, because the CMC coating covered on sample hindered oxidative decomposition. The content of cynaroside in cherry was significantly increased by US-IMO pretreatment as compared to the control. This probably because US combined with IMO osmotic pretreatment, the IMO solution penetrated into the tissue, enriched the cells, and improved the content of cynaroside. The highest catechin and kaempferol content was found in cherry after the US-CMC pretreatment (59.07 ± 2.05 m g/100 g and 12.42 ± 0.23 mg/100 g).

**Table 2:** The natural active substances content of dried cherries after US-CMC, US-CE, US-EA, US-IMO, and US-(PC + AEEO) pretreatment.

Active substances (mg/100 g)	Control	US-CMC + RFV	US-CE + RFV	US-EA + RFV	US-IMO + RFV	US-(PC + AEEO) + RFV
Catechin	38.44 ± 2.33^e^	59.07 ± 2.05^a^	54.28 ± 1.12^b^	49.27 ± 3.07^c^	51.44 ± 2.29^c^	43.28 ± 3.15^d^
Cynaroside	25.49 ± 1.49^c^	36.25 ± 1.08^a^	30.48 ± 2.17^bc^	31.44 ± 1.06^b^	36.98 ± 1.84^a^	29.47 ± 2.01^bc^
Quercetin	10.49 ± 1.00^d^	18.24 ± 0.45^a^	18.01 ± 0.89^a^	17.45 ± 1.07^ab^	12.14 ± 0.79^c^	16.57 ± 0.48^b^
Kaempferol	9.48 ± 0.29^d^	12.42 ± 0.23^a^	11.74 ± 0.17^b^	10.41 ± 0.24^c^	11.10 ± 0.13^bc^	11.07 ± 1.05^bc^
Chlorogenic acid	230.17 ± 5.22^e^	301.22 ± 7.10^a^	288.49 ± 5.78^b^	269.48 ± 9.27^d^	265.44 ± 4.56^d^	277.48 ± 7.44^c^
Neochlorogenic acid	192.48 ± 3.78^e^	233.45 ± 3.55^b^	241.48 ± 4.12^a^	221.11 ± 1.97^c^	215.45 ± 2.89^d^	216.47 ± 3.04^d^
Cyanidin-3-O-rutinoside	12.26 ± 0.81^d^	20.89 ± 0.64^a^	18.18 ± 0.75^b^	16.78 ± 0.62^c^	15.44 ± 0.37^cd^	15.79 ± 0.56^cd^
Isochlorogenic acid	16.10 ± 2.01^d^	23.44 ± 1.75^a^	23.79 ± 1.03^a^	19.44 ± 2.04^c^	21.75 ± 1.25^b^	19.22 ± 0.94^c^

Anthocyanins, as a water-soluble natural pigment, are the main characteristic chemical compounds that cause color changes in cherry, with cyanidin-3-O-rutinoside playing a dominant role. The cyanidin-3-O-rutinoside content of cherry after US-CMC, US-CE, US-EA, US-IMO and US-(PC + AEEO) pretreatments were 20.89 ± 0.64, 18.18 ± 0.75, 16.78 ± 0.62, 15.44 ± 0.37 and 15.79 ± 0.56 mg/100 g, respectively. In comparison to the control, the cyanidin-3-O-rutinoside content increased by 70.39 %, 48.29 %, 36.87 %, 25.94 % and 28.79 %, respectively. The reason may be that cyanidin-3-O-rutinoside was highly sensitive compounds to degradation by heat, light and oxidation during the dehydration process at high temperature and high humidity for a long period of time. US combined with chemical pretreatment is beneficial in preventing thermal and oxidative damage to bioactive compounds and reducing the loss of heat-sensitive nutrients. A similar result was reported in the case of chestnut by Fijalkowska et al. [58]. Overall, the US-CMC and US-CE coating pretreatments resulted in better retention of the natural active substances in cherries.

### 3.6 Soluble solid

The soluble solid content (SSC) is a critical determinant of the inherent quality of cherries. The soluble solids content of cherries after the different pre-treatments ranged from 11.69 ± 0.72 to 13.79 ± 0.68 °Bx and increased by 19.41–40.86 % compared with the control (Table 1). The SSC in cherries after US-CMC and US-CE pretreatments were 13.79 ± 0.68 and 13.14 ± 0.43 °Bx, respectively, which were higher than the other pretreatments significantly. This suggested that the utilization of US combined with CMC or CE coatings had a synergistic effect, and can better protect the SSC in cherries [59]. Correlation analysis shown that the soluble solid content displayed positive correlations with TPC (r = 0.87, *P* < 0.05), TFC (r = 0.91, *P* < 0.01), and natural active substances content (r = 0.75–0.97, *P* < 0.01), (Fig. 7(C)). Higher soluble solid content was also observed in dried cherry products after US + EA pretreatment. No significant differences in SSC between US-IMO and US-(PC + AEEO) samples was observed. This probably because IMO and PC + AEEO solution could induce the severe cell collapse and then SSC was leaking from the cells.

**Fig. 5:**
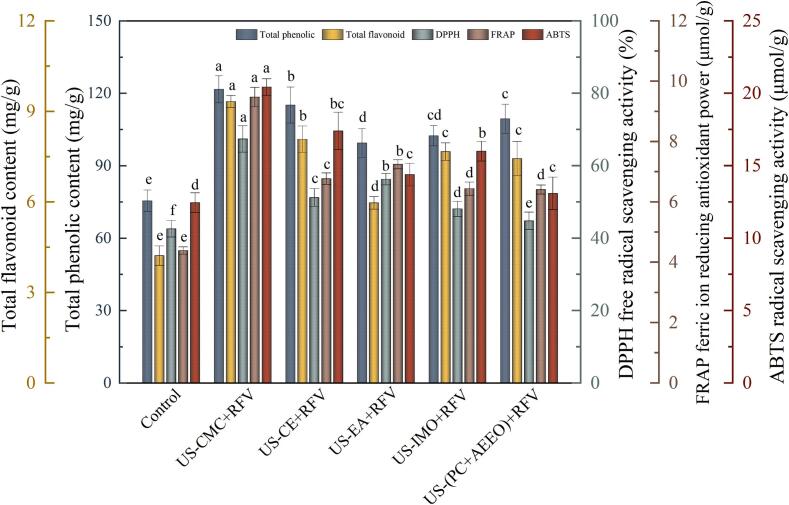
TPC, TFC, and antioxidant activity (DPPH, ABTS, FRAP) contents of cherries subjected to various pretreatment conditions.

**Fig. 6:**
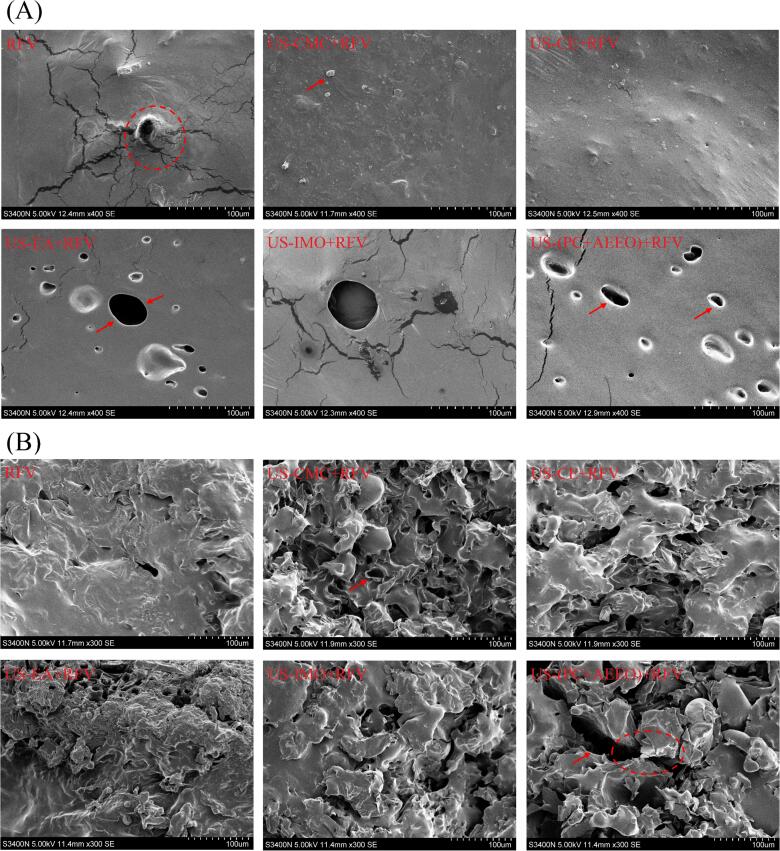
Surface (A) and internal (B) microstructure of cherries subjected to various pretreatment conditions.

**Fig. 7:**
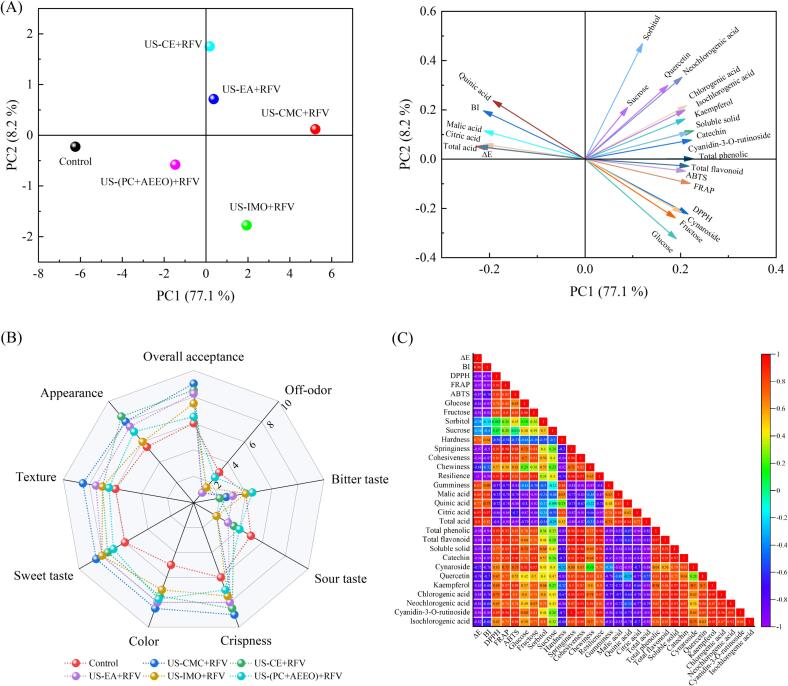
PCA (A), sensory evaluation (B) and correlation analysis (C) of quality properties of cherries observed from various pretreatment methods.

### 3.7 Texture

Texture represents the sensory expression of the matrix and structural attributes of food, while the textural characteristics of cherries exert a direct impact on their gustatory perception and flavor profile, thereby serving as a fundamental sensory criterion for assessing product quality. The texture properties of cherry subjected to various treatments are presented in Table 3. ANOVA results revealed a significant influence of varied pretreatments on the texture properties of cherries (*P* < 0.05). The samples RFV obtained the highest values of cohesiveness (0.69 ± 0.12), springiness (82.33 ± 4.28 %), and resilience (36.79 ± 3.07 %) after US-CMC pretreatment, which could be because the CMC was beneficial to maintaining the cytoskeleton, thereby reducing shrinkage and increasing texture properties. Similar results were observed in the results of the US-CE pretreatment. The US-CE + RFV dried samples exhibited the highest chewiness value (7.48 ± 0.19N), followed by US-CMC + RFV (6.31 ± 0.21N), whereas the least value was observed in the control sample (4.54 ± 0.12N).

**Table 3:** The texture of dried cherries after US-CMC, US-CE, US-EA, US-IMO, and US-(PC + AEEO) pretreatment.

Drying conditions	Hardness (N)	Springiness (%)	Cohesiveness	Chewiness (N)	Resilience (%)	Gumminess (N)
Control	20.45 ± 0.48^a^	60.28 ± 3.79^e^	0.31 ± 0.09^d^	4.54 ± 0.12^e^	23.48 ± 2.44^f^	8.34 ± 0.11^a^
US-CMC + RFV	15.44 ± 0.21^c^	82.33 ± 4.28^a^	0.69 ± 0.12^a^	6.31 ± 0.21^b^	36.79 ± 3.07^a^	4.26 ± 0.07^d^
US-CE + RFV	12.48 ± 0.33^d^	74.33 ± 4.01^b^	0.64 ± 0.08^ab^	7.48 ± 0.19^a^	31.48 ± 1.75^c^	3.79 ± 0.12^e^
US-EA + RFV	13.19 ± 0.19^cd^	72.48 ± 3.22^c^	0.58 ± 0.11^b^	6.22 ± 0.23^b^	33.29 ± 1.07^b^	5.69 ± 0.08^c^
US-IMO + RFV	17.68 ± 0.24^bc^	68.48 ± 2.14^d^	0.50 ± 0.04^c^	4.72 ± 0.09^d^	30.07 ± 2.05^d^	7.48 ± 0.04^bc^
US-(PC + AEEO) + RFV	18.22 ± 0.40^b^	69.22 ± 3.77^d^	0.47 ± 0.16^cd^	5.10 ± 0.17^c^	28.49 ± 2.44^e^	7.97 ± 0.16^b^

As shown in Table 3, the hardness value followed a rank order: Control > US-(PC + AEEO) > US-IMO > US-CMC > US-CE > US-EA, which were substantial decreased by 10.90 %–38.97 % after pretreatments. Because the cavitation effect of US, more microfine channels were generated inside the cherry tissues resulting in a decrease in the compactness of the tissue structure and a reduction in the hardness of the dehydrated cherries. The US-IMO pretreatment significantly increased the hardness of cherries compared to the US-EA, probably due to the hydroxyl groups of sugar molecules penetrated into the samples tissue interacting with water molecules to form hydrogen bonds, resulting in the internal structure of the raw material's tissue being denser and the loss of elasticity. In addition, part of the sugar molecules attached to the surface of the sample may crystallize under heating conditions, inducd the occurrence of crusting phenomenon on the surface of the raw material and increasing its hardness. The US-(PC + AEEO) treatment samples also exhibited hardness values similar to those of US-IMO. It was possible that US-(PC + AEEO) + RFV had a shorter drying time, resulting in severe cherry shrinkage and surface hardening phenomenon. Notably, some researchers found that loss of hardness favored a softer flavor [40].

### 3.8 Total phenolic and total flavonoid

Phenolics and flavonoids are important secondary metabolites and play an important role in the antioxidant process in plants. They are also important antioxidant active components in cherries, which can effectively scavenge active oxygen free radicals and inhibit membrane lipid peroxidation. Significant effects were observed on the TPC and TFC of cherries following various pretreatment (*P* < 0.05). The TPC and TFC of the dried cherry products ranged from 75.48 ± 4.48 to 121.67 ± 5.59 mg/g and 4.22 ± 3.32 to 9.33 ± 0.48 mg/g, respectively, after the five pretreatments (Fig. 5). The highest TPC and TFC were found in the US-CMC pretreated sample varieties with 121.67 mg/g and 9.33 mg/g, respectively. The possible reason was that the internal tissue cell structure of cherry was destroyed during US pretreatment promoted the release of phenolic and flavonoid compounds, and the CMC edible coating hindered the oxidative degradation of heat-sensitive substances during the RFV process [60], [61]. TPC were increased by 45.01 % and 35.73 % after US-(PC + AEEO) and US-IMO pretreatments, respectively, compared to the control (75.48 ± 4.48). The main reason was that TPC and TFC were susceptible to oxygen, light and heat, but US-(PC + AEEO) and US-IMO pretreatments shorten the drying time and inhibit the polyphenol oxidase (PPO) and peroxidase (POD) activities of cherries after dehydration.

The corresponding TFC after US-CE, US-EA, US-IMO and US-(PC + AEEO) pretreatments were 8.08 ± 0.44, 5.97 ± 0.21, 7.67 ± 0.29, and 7.44 ± 0.57 mg/g, representing increments of 91.47 %, 41.47 %, 81.75 %, and 73.30 % compared to the control. These findings suggest that US coupled with chemical reagent treatment may attenuate the biodegradation of nutrient substances to a certain extent. On the one hand, ultrasound's mechanical and cavitation effects enhanced the heat and mass transfer efficiency during dehydration, mitigated the oxygen content and cellular tissue sensitivity in cherries, and attenuated the oxidative degradation of phenolic compounds. Comparable outcomes were documented in the investigation conducted by Yao et al [62]. On the other hand, US combined with various chemical pretreatments improved the retention of bioactive compounds in cherry by blunting enzyme activity and destroying the wax layer on the surface of the material.

### 3.9 Antioxidant capacity

Antioxidant activity is an important indicator for evaluating the nutritional value and functional properties of fruits and vegetables. Studies have shown that the antioxidant activity of cherries is mainly derived from the antioxidant enzyme system and non-enzymatic bioactive substances in the body, such as ascorbic acid, TPC, TFC, and anthocyanosides [63]. The antioxidant capacity of cherry under different pretreatment conditions are shown in Fig. 5. The DPPH and ABTS of cherries pretreated with US-CE, US-CMC, US-EA, US-IMO and US-(PC + AEEO) increased by 20.32 %, 58.37 %, 32.21 %, 12.92, 5.17 %% and 39.84 %, 64.18 %, 15.58 %, 28.59 %, 5.14 %, respectively, compared to the control. The free radicals generated by US action may interact with the aromatic rings of phenolics and contribute to the antioxidant capacity of bioactive substances. Moreover, pretreatment may disrupt covalent bonds, causing cells to release antioxidants such as phenolics, flavonoids and carotenoids. Research has also reported a highly positive correlation between phenolic compounds and antioxidant activity [64]. Furthermore, pearson correlation analysis also showed that antioxidant activity and TPC (r = 0.93, *P* < 0.01) exhibit a higher correlation than TFC (r = 0.89, *P* < 0.05). The FRAP of the five pretreatments cherries were determined and the highest antioxidant activity was found in the US-CMC samples, while the US-(PC + AEEO) pretreatment samples showed the lowest FRAP values. The possible reason was that the CMC coating on the surface of the cherries hindered oxygen and eased the loss of heat-sensitive compounds during thermal treatment. A comparable phenomenon was also discovered and elucidated by Salehi et al. [54] in their investigation of cherries. At the same time, the release of phenolic substances from the cell matrix was increased by US. Additionally, US-EA also showed better antioxidant capacity, which may be attributed to the fact that EA, as an organic solvent, combined with US can slow the degree of oxidative degradation and passivate the PPO and POD enzymes in cherry, thereby inhibiting the oxidative condensation of polyphenolic compounds during the drying process. The FRAP ferric ion reducing antioxidant power in cherries were 6.44 ± 0.22 and 6.41 ± 0.15 μmol/g after US-(PC + AEEO) and US-IMO treatments, respectively, which were not significantly different (*P* > 0.05). This probably because IMO and (PC + AEEO) solution could induce the severe cell collapse and then increase the probability of adverse reactions such as oxidation, therefore the antioxidant properties of the samples were lower.

### 3.10 Microstructure analysis

Changes in the organizational structure of the material during the drying process play an important role in the internal moisture diffusion characteristics and heat and mass transfer efficiency. The microstructure of the surface and interior of dried cherry products was examined and analysied using scanning electron microscopy, to further investigate the effect of US combined with chemical pretreatment on the microstructure and moisture diffusion of cherries. The RFV (control) sample showed a high density, less-organised and irregular cell structure, which appeared to be broken and collapsed. Meanwhile, as the sample was exposed to high temperature and high humidity for a long time, part of the cells were crumpled and closed due to capillary stress, which reduced the capillary pore channels required for water diffusion and flow, resulting in the intensification of the surface hardening and the reduction of the heat-mass transfer efficiency. Compared with the control, the sample tissues after US combined with chemical pretreatment produced a loose and porous structure as well a clear cell wall, indicating that the application of US can make the microchannels formed in the internal tissues of the samples and then accelerated the heat and mass transfer and the diffusion of water inside the samples. This may be due to the high-frequency oscillations between molecules caused by mechanical effect of US, the micro-capillary of the material were extruded and sheared, to expand the original microcapillary. The cavitation effect can produce micro-bubbles and micro-jet, micro-bubble bursting of the organizational structure of the strong impact and destruction, resulting in new micro-pore channels. This finding corroborated that the application of US had a positive impact on improving the drying rate and reducing energy consumption. Furthermore, samples US-CMC + RFV and US-EA + RFV showed a looser cell structure than other sample, which could be because the CMC, as a hydrocolloid coating material, was beneficial to maintaining the cytoskeleton, thereby reducing shrinkage. EA has the ability to dissolve cell wall components, which can change the cell structure to increase its permeability when drying, EA is rapidly vaporized by heat, and volume expansion, so that the internal pore structure of the material increases. Therefore, combined pretreatments can better protect the internal organizational structure of cherries. However, the internal tissue structure of the cherries was damaged to a higher extent after US-(PC + AEEO) treatment, which could be attributed to the rapid water loss of the US-(PC + AEEO)-treated samples at the early stage of drying, leading to the deformation of the tissue structure and the generation of localized stresses. When the stresses accumulated to exceed the bonding force between the tissue structures, it caused the tearing and collapse of the microporous channels.

### 3.11 Sensory evaluation

Sensory evaluation researches are inevitable to choose appropriate drying techniques and to optimize the process of dehydration. Good sweet and sour taste, crispness, aroma, appearance, texture and overall acceptance are important features of high-quality fruit. However, bitterness and off-odor are considered unfavorable qualities in cherry fruits. The representative sensory properties of dried cherry at various treatments are shown in Fig. 7(B). The overall acceptance scores of the cherries after US-CMC pretreatment (9.0 points) ranked the highest, followed by US-CE (8.5 points) and US-EA pretreatment (8.25 points), with the control scoring the lowest (6.0 points). Evidently, US-CMC + RFV dried cherries were more appreciated and preferred by consumers. Our data suggest that bitter taste was significantly different between control and prepretreateds (*P* < 0.05). Dried cherry products had a strong bitter taste after US-(PC + AEEO) pretreatment, because US-(PC + AEEO) treatment caused more limocitrin-7-O-glucoside, plant-derived amino acids, and alkaloids to be released from the matrix in the samples [1]. Concurrently, the high concentration of lye treatment caused some lye to remain on the surface of the sample after drying, resulting in a salty and bitter taste of the cherries. Cherries subjected to US-CMC and US-CE pretreatments exhibited better color attributes. The reason was that the US combined with CMC and CE coating reduced the synergistic effects of phenolic substrates, oxygen, and enzymes, slowed down enzymatic browning, pigment degradation, and the Maillard reaction [65]. Changes in appearance caused by cherry drying are mainly manifested as a reduction in volume shrinkage, which can seriously degrade the sensory quality of the samples. Among the five pretreatments, a minimum appearance score was observed in the US-IMO + RFV-dried cherry, due to the high concentration of IMO solution and high-intensity US led to the internal and external pressure balance, disruption of the original tissue structure, and the formation of shrinkage stress, resulting in wrinkling. The analytical findings indicated the absence of off-odor in the cherry subsequent to pretreatment. The off-odor score in the control was 3.0, which was related to the dissipation and degradation of volatile components due to prolonged high-temperature drying.

### 3.12 Principal component analysis (PCA) and correlation analysis

PCA and correlation analysis were applied to analyse the interrelationships between physicochemical properties under various pretreatment methods (Fig. 7(A)). Among them, PC1 and PC2 were 77.1 % and 8.2 % (85.3 % in total), respectively. This indicated that PC1 and PC2 could describe the relationship between the quality attributes of cherries and dehydration techniques. The dominant variables in PC1 were soluble solid (0.359), catechin (0.329), neochlorogenic acid (0.252), total phenolic (0.249) and Total flavonoid (0.257), while in PC2, mainly variables were cynaroside (0.469), glucose (0.508), and fructose (0.359). Results showed that US-CMC and US-EA were located on the right side of the score plot, indicating these two pretreatments and especially US-CMC could better retained the quality properties of cherries.

## 4 Conclusion

The objective of this investigation was to assess the effect of US combined with chemical pretreatment on drying characteristics, quality properties, texture attributes, and sensory evaluation of RFV dried cherries. The results indicated that the application of US combined with chemical pretreatment could increase the dehydration rate and a substantial reduction in drying time and energy consumption during the RFV drying process. Compared with other dried samples, US-CMC + RFV dried cherry (ΔE = 5.59 ± 1.01 and BI = 21.77 ± 2.49) obtained the closest color attributes to fresh samples. This is because US-CMC treatment inhibited the activity of PPO and POD to a certain extent, which reduced the degradation of phenolic substances. Significant positive correlations between free radical scavenging rates of DPPH and FRAP and the content of TPC, soluble solids and phenolic acids were demonstrated (*P* < 0.05). Furthermore, US-CMC pretreatment exhibited better preservation of physicochemical qualities in all dried products. As expected, compared with untreated sample, the texture properties and sensory evaluation of cherries treated with US-CMC and US-CE exhibited superior performance. This demonstrated that the application of US combined with edible coatings pretreatment could dramatically enhance the overall acceptance of cherries. This study provides valuable reference for the application of US combined with chemical pretreatment, especially US combined with edible coatings, in the dehydration of fruits and vegetables and demonstrates their potential to protect bioactive compounds in food materials effectively.

## CRediT authorship contribution statement

**Zepeng Zang:** Writing – original draft, Validation, Software, Methodology, Investigation, Formal analysis, Data curation, Conceptualization. **Fangxin Wan:** Writing – review & editing, Validation, Supervision, Methodology, Conceptualization. **Guojun Ma:** Writing – review & editing, Supervision, Formal analysis, Conceptualization. **Yanrui Xu:** Validation, Methodology, Investigation, Conceptualization. **Bowen Wu:** Visualization, Software, Investigation, Data curation, Conceptualization. **Xiaopeng Huang:** Writing – review & editing, Visualization, Resources, Methodology, Funding acquisition, Conceptualization.

## Declaration of competing interest

The authors declare that they have no known competing financial interests or personal relationships that could have appeared to influence the work reported in this paper.
